# Prediction of treatment response and survival in patients with advanced hepatocellular carcinoma receiving sorafenib: pretreatment prognostic scores

**DOI:** 10.1186/s43046-026-00417-z

**Published:** 2026-10-09

**Authors:** Ammar Hatem, Mohamed Mahmoud Nabeel, Samar K Darweesh, Rania Zayed, Ashraf Omar Abdelaziz, Hend Ibrahim Shousha, Ahmed Hosni Abdelmaksoud, Eman Medhat, Tamer Mahmoud Elbaz, Mennatallah Ali Rashwan, Ahmed Ramadan, Rania Lithy

**Affiliations:** 1 Endemic Medicine Department, Faculty of medicine, Cairo University, Giza, Egypt; 2 Clinical and Chemical Pathology Department, Faculty of Medicine, Cairo University, Giza, Egypt; 3 Diagnostic and Interventional Radiology Department, Faculty of Medicine, Cairo University, Giza, Egypt

**Keywords:** Hepatocellular carcinoma, Sorafenib, Predictors, Survival

## Abstract

**Introduction:**

Sorafenib is currently among the recommended first-line medications for patients with advanced hepatocellular carcinoma (HCC); however, response to sorafenib remains unpredictable despite efforts to define prognosis. This study aims to predict treatment response and survival in patients with sorafenib-treated HCC using neutrophil-to-lymphocyte ratio (NLR), albumin-bilirubin (ALBI) grade, CRAFITY score, and Glasgow prognostic score (GPS).

**Methods:**

This prospective study recruited 75 Egyptian patients with Sorafenib-treated HCC. Pretreatment laboratory parameters and imaging studies were obtained. The neutrophil-to-lymphocyte ratio, ALBI grade, CRAFITY score, and GPS were calculated for each patient. All the patients were followed for treatment response, overall survival, and sorafenib tolerance.

**Results:**

Most of the participants were males, with a median age of 62 years. HCV was the most common underlying cause. Most of the patients had a low pretreatment NLR (77.33%), grade 2 ALBI (65.33%), 41.89% had a high CRAFITY score, and 48.6% had GPS 1. Patients were divided into two groups: group 1: sorafenib-naïve (*n* = 44) and group 2: patients with previous interventional therapy (*n* = 31). Pretreatment AST, ALT, mild ascites, portal invasion, and BCLC stage C were significantly higher among sorafenib-naïve patients. Median OS and time to progression for the entire cohort were 4 months and 9 months, respectively. No significant differences were observed in terms of OS between various NLR, ALBI grades, CRAFITY scores, and GPS. Multivariate analysis showed a high risk of mortality among sorafenib-intolerant patients (*p* = 0.01, HR: 2.33, 95% CI: 1.23–4.42).

**Conclusion:**

Overall survival and time to progression are not affected by different pretreatment values of NLR, ALBI grade, CRAFITY score, and Glasgow prognostic score in sorafenib-treated HCC.

## Introduction

Worldwide, hepatocellular carcinoma is a significant health problem, accounting for 7% of all cancers and constituting approximately 90% of primary liver cancers [1]. Hepatocellular carcinoma was considered the fifth most frequent cancer in males and the ninth most common cancer in females, in addition to being the second leading cause of cancer-related death [2]. The majority of hepatocellular carcinoma patients have underlying liver cirrhosis resulting from known aetiological risk factors, such as infection with the hepatitis C or hepatitis B viruses, alcohol-related liver disease (ALD), or metabolic dysfunction-associated steatotic liver disease (MASLD) [3].

Although diagnostic and screening methods have evolved, most HCC patients are found to have advanced disease (BCLC stage C), making them ineligible for locoregional or surgical interventions [4]. Furthermore, about 30% of HCC patients have had intermediate-stage disease (BCLC stage B). Although TACE is commonly regarded as the first-line treatment for intermediate-stage HCC, there is evidence that systemic therapy could be considered in patients with large tumor burden, bilobar or infiltrative tumors and in disease that is progressing after arterial-directed therapy [5].

The approval of sorafenib by the FDA in 2007 dramatically changed the landscape of HCC management [6]. The median overall survival of patients treated with sorafenib has improved from 10.7 months in the SHARP study (2005–2006) [7], to 12.3 months in the REFLECT study (2015) [8] and over 13 months in the IMbrave-150 study (2018–2019) [9]. Although sorafenib is currently recommended as a first-line therapy for patients with BCLC stage C hepatocellular carcinoma (HCC), treatment outcomes remain unpredictable despite ongoing efforts to establish prognosis based on clinical staging [10].

Hepatocellular carcinoma is an inflammation-associated type of cancer, as more than 90% of HCC cases are caused by hepatic damage and persistent inflammation [11]. Several studies showed that elevated baseline NLR was significantly associated with worse OS in HCC patients treated with Sorafenib [12]. The median OS for patients with HCC after treatment with sorafenib was 5.23 months and 10.05 months for patients with pretreatment NLR > 3.5 and NLR < or equal to 3.5, respectively [13]. Albumin to bilirubin grade 1(ALBI grade 1) was linked to improved progression-free survival in patients with advanced HCC receiving sorafenib. In the study by Kuo et al., the OS for ALBI grades 1, 2 and 3 after sorafenib therapy significantly decreased from 14.8 months to 5 and 0.7 months, respectively [14].

Although the CRAFITY score (C-reactive protein and alpha-fetoprotein in immunotherapy) was associated with survival, in patients receiving sorafenib, it was not able to predict the rate of disease control. It was shown that the CRAFITY-low group had a median overall survival of 18.3 months, the CRAFITY-intermediate group of 7.9 months, and the CRAFITY-high group of 4.6 months [15]. It has been demonstrated that the Glasgow prognostic score significantly predicts overall survival and time to progression in patients receiving sorafenib therapy for advanced HCC [16]. The present study aims to predict treatment response and survival in patients with sorafenib-treated hepatocellular carcinoma using the following pretreatment scores/grades: Neutrophil-to-lymphocyte ratio, Albumin-bilirubin grade, and CRAFITY score.

## Patients and methods

This is a prospective cohort study that included 75 Egyptian patients with advanced hepatocellular carcinoma who were treated with sorafenib. Patients were recruited from the HCC multidisciplinary clinic, Endemic Medicine Department, during the period from August 2022 to April 2024. The study protocol was approved by the Ethical Committee of the HCC multidisciplinary clinic on 29th of August 2022 (code: MD-240-2022).

The eligibility criteria were an adult patient with preserved liver function, CTP A5-6, who had hepatocellular carcinoma, BCLC stage C and/or BCLC stage B with extensive/infiltrating disease not amenable to loco-regional therapy or progressing after local/loco-regional therapy. Patients with Child-Turcotte-Pugh score more than A6, performance status more than 2 based on ECOG score, concomitant malignancies other than HCC, and Patients with clinically evident infection or receiving steroid therapy were excluded from our study.

All the recruited patients were assessed clinically and by laboratory investigation before sorafenib administration. Full blood count (CBC), neutrophil and lymphocyte percentages, alanine aminotransferase (ALT), aspartate aminotransferase (AST), total bilirubin, serum albumin, serum creatinine, alpha-fetoprotein (AFP), C-reactive protein (CRP), international normalized ratio (INR), and viral markers were obtained before therapy.

Hepatocellular carcinoma was diagnosed in accordance with the international guidelines [17, 18]. HCC was staged according to the Barcelona Clinic Liver Cancer (BCLC) classification. Evaluation of the severity of liver disease was done using the modified Child-Pugh score and the performance status was assessed using the Eastern Cooperative Oncology Group (ECOG) performance status scale. The response of the tumour was assessed using the modified Response Evaluation Criteria in Solid Tumours guideline (mRECIST) criteria as follows: complete response (CR): no contrast enhancement inside the lesion on CT in the arterial phase; partial response (PR): > or equal to 30% decrease in the sum of all target lesions in the longest axis measurement; progressive disease (PD): >20% increase in viable target lesions; and stable disease: non-PR and non-PD [19].

In addition to tumor-related characteristics, the prognosis of HCC is highly dependent on several additional factors arising from the underlying chronic liver disease. As hepatocellular carcinoma is an inflammation-associated type of cancer occurring in a patient with underlying liver disease, several prognostic scoring systems reflecting liver function, systemic inflammation or immunonutrition status were assessed to predict treatment response and survival: ALBI grades, CRAFITY score, NLR, and GPS [20].

Neutrophil-To-Lymphocyte Ratio (NLR) was calculated based on the ratio of absolute neutrophil count divided by absolute lymphocyte count. Patients were divided into a high NLR group (> 3.53) and a normal NLR group (equal or less than 3.53) [21]. The albumin–bilirubin (ALBI) score is calculated using serum albumin and total bilirubin values according to the following formula: ALBI score = (log10 bilirubin [µmol/L] × 0.66) + (albumin [g/L] × − 0.085). Based on this score, patients are classified into grade 1 (≤ − 2.60), grade 2 (–2.59 to − 1.39), or grade 3 (> − 1.39) [14]. CRAFITY score was calculated based on baseline Alpha-fetoprotein and C-reactive protein and graded into: 0 points: AFP < 100 ng/ml and CRP < 10 mg/l, 1 point: either AFP ≥ 100 ng/ml or CRP ≥ 10 mg/l, and 2 points: AFP ≥ 100 ng/ml and CRP ≥ 10 mg/l [15]. Glasgow prognostic score was calculated based on C-reactive protein and serum albumin level and graded into three scores; A score of 2 was assigned to individuals who had both hypoalbuminemia (< 3.5 g/dl) and an increased C-reactive protein level (> 1.0 mg/dl); patients who had just one or neither of these biochemical abnormalities received a value of 1 or 0, respectively [16].

All enrolled participants then started sorafenib therapy (200 mg once daily, increased to 200 mg twice daily, then increased to 400 mg twice daily if well tolerated). All the recruited patients were followed initially every week for two weeks and at one-month intervals, then every three months. With each visit, all the patients were assessed clinically and with laboratory investigations for sorafenib adverse events and decompensating manifestations. Sorafenib intolerance was defined, classified, and managed based on the NCCVH guideline for the use of systemic therapies in patients with HCC. Sorafenib dose modification, interruption, or permanent discontinuation was applied in patients who developed adverse events based on the type and severity of the events.

The treatment response of HCC patients was evaluated by the modified RECIST criteria every three months. Treatment was continued until hepatic decompensation, disease progression, Unacceptable side effects, or death. The time to progression (TTP): The time from initiation of sorafenib therapy to disease progression was assessed for all patients who completed at least three months of therapy, while overall survival was calculated for all patients from the date of enrollment until study completion, death, discontinuation or the last follow-up visit. Survival curves were plotted by the Kaplan-Meier method and compared using the log-rank test for all patients and for various results of neutrophil-to-lymphocyte ratio, ALBI grades, CRAFITY, and Glasgow prognostic scores. Independent prognostic factors were estimated by the Cox proportional hazards model in univariate and multivariate regression models.

**Fig. 1:**
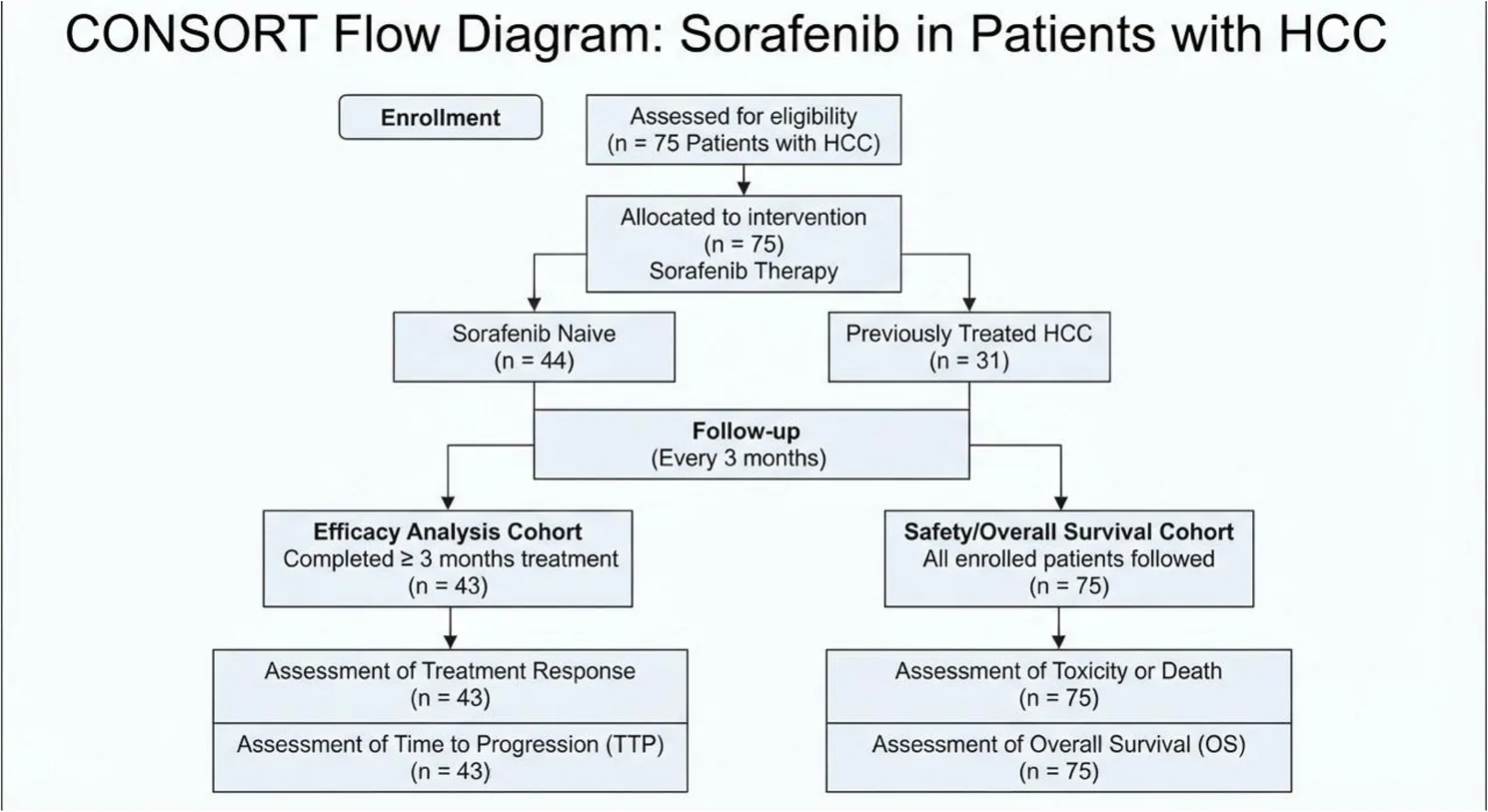
The CONSORT flow diagram: sorafenib in patients with HCC

We divided our Patients according to the history of previous interventional therapy of the same lesion into: Group 1: Sorafenib-naïve, no previous interventional therapy (*n* = 44) and Group 2: Previously treated patients (*n* = 31). Both groups were compared according to patients’ characteristics, laboratory parameters, hepatocellular carcinoma characteristics, pretreatment predictive scores, treatment response based on modified RECIST, Sorafenib adverse events, and survival and progression analysis, as shown in (Fig. 1).

### Statistical analysis

The statistical package for the social sciences (SPSS) version 26 was used to code and enter the data (IBM Corp., Armonk, NY, USA). For quantitative data, the mean, standard deviation, median, minimum, and maximum were used, while categorical data were summarized using frequency (count) and relative frequency (%). The nonparametric Mann-Whitney test was used to make comparisons between quantitative variables. The chi-square test was used to compare the categorical data. When the expected frequency is less than 5, the exact test was used. The Spearman correlation coefficient was used to calculate correlations between quantitative variables. A survival curve was plotted by the Kaplan-Meier method. P-values less than 0.05 were considered statistically significant.

## Results

This is a prospective cohort study that included 75 patients with advanced HCC treated with Sorafenib attending the HCC multidisciplinary clinic, Endemic Medicine Department, Kasr-Alainy Hospital, Cairo University during the period from August 2022 to April 2024.

The median age of the studied patients was 62 years, with male predominance (74.67%). Family history of HCC was present in 4% of the studied population. Chronic HCV was the most common cause of the underlying liver disease (86.7%), and most of them received DAAs and achieved SVR. There was a mild increase in the median AST, a mild increase in the median CRP, and the median AFP value was 484.0. The median MELD score of the studied population was 9.28. Regarding the Child-Pugh score, 42 patients had Child-Pugh class A5 liver cirrhosis, while 31 patients were in Child-Pugh class A6, and only two patients were non-cirrhotic. Most of the patients had performance status 1 (*n* = 46, 62.2%), while 26 patients (35.1%) had performance status 0, and two patients (2.7%) had performance status 2 (Table 1). Portal vein invasion was detected in 45.33%, lymph node involvement in 48% and tumor metastases in 18.84% of the studied population. The median tumor size was 6 cm (5–9), most lesions were multiple (45.9%) and were in the right hepatic lobe (54.05%). Regarding the BCLC staging system, 78.67% were in BCLC stage C and 21.33% were in BCLC stage B (Table 1).

**Table 1:** Baseline demographic, laboratory and radiologic features of the studied population

Variable					Number	(Percentage)
Age in years Median (Min-Max)					62 (25–79)	
Sex			Female		19	(25.3%)
			Male		56	(74.7%)
Cigarette Smoking					24	32.0%
Diabetes mellitus					13	17.3%
HCC Family history					3	4%
Underlying liver disease				HBV	2	(2.7%)
				HCV	65	(86.7%)
					DAAs intake 53	70.66%
					SVR 49	92.45%
				unknown	8	(10.7%)
Non cirrhotic					**2**	2.67%
Cirrhotic					**73**	97.33%
Child-Pugh score				A5	42	56%
				A6	31	41.3%
Performance status. 0					26	35.14%
1					46	62.16%
2					2	2.70%
					**Median**	**Range**
Haemoglobin (13–17 g/dl)					12.50	9-17.3
WBCs** (4-11 × 10**^**3**^**/ ul)**					6.10	2.7–13.2
Neutrophil %					65.0	(40.0–86.0)
Lymphocyte %					27.0	(11.0–45.0)
Platelets (150-450 ×** 10**^**3**^**/ul)**					165	(55–375)
ALT (up to 41 IU/ L)					29.0	(8.0- 188.0)
AST (up to 38 IU/L)					45.0	(7.0- 211.0)
Bilirubin (1-1.2 mg/dl)					0.90	(0.26- 2.00)
Albumin 3.5-5 gm/dl)					3.70	(2.90–5.30)
INR					1.17	(0.39–1.64)
Serum creatinine **(0.2–1.2 mg/dl)**					0.87	(0.40–1.50)
C-reactive protein < 10 mg/dl					11.1	(0.6- 356.7)
AFP (10–20 ng/ml)					484.0	(1.3- 79796.0)
MELD score					9.28	6–15
Size of the largest lesion					6	5–9
					**Count**	**Percentage**
Number of hepatic focal lesions.			**1**		33	44%
			**2**		7	9.33%
			Multiple		34	45.9%
Site of hepatic focal lesion(s).	Right lobe				40	54.05%
	Left lobe				9	12.16%
	Both lobes				25	33.78%
Mild ascites					6	8%
Portal vein.		Patent			41	54.67%
		Thrombosed			34	45.33%
Lymph nodes involvement					36	48%
Metastases					13	18.84%
BCLC stage				B	16	21.33%
				C	59	78.67%

*WBCs* white blood cells, *ALT* alanine transaminase, *AST* aspartate transaminase, *INR* international normalized ratio, *AFP* alpha-feto protein, *MELD score* model for end stage liver disease, *BCLC* Barcelona: Barcelona Clinic Liver Cancer

Neutrophil-to-lymphocyte ratio was assessed in 73 patients, ALBI grade in 75, CRAFITY score in 74, and Glasgow prognostic score in 74 patients. Most of the patients had a low neutrophil – lymphocyte ratio (77.33%), grade 2 ALBI (65.33%), Glasgow prognostic score 1(48.6%), and 41.89% had a high CRAFITY score versus 39.19% intermediate and 18.92% low CRAFITY score (Table 2).

**Table 2:** Pretreatment predictive scores

Variable		Count	Percentage
Neutrophil-lymphocyte ratio	Low	58	77.33%
	High	15	20%
ALBI score	**1**	25	33.33%
	**2**	49	65.33%
	**3**	1	1.33%
CRAFITY score	Low	14	18.92%
	Intermediate	29	39.19%
	High	31	41.89%
Glasgow prognostic score	**0**	25	33.8%
	**1**	36	48.6%
	**2**	13	17.6%

Various types of sorafenib adverse events were seen. The most common adverse event reported in our study was hepatic decompensation with ascites in 30.7% of the studied population, followed by increased liver enzymes (20.0%), hematemesis (6.7%) and severe HFSR (6.7%). Treatment response according to modified RECIST was assessed in 43 patients who completed at least 3 months of sorafenib therapy. Progressive disease was the most common (38.7%), followed by stable disease in 17.3%. By the end of the study, 87.7% of the population had died. The median survival time was 4 months (1–20) and the median post-sorafenib survival, after cessation of treatment due to either progression or intolerance, was 1.5 months (0–15) (Table 3).

**Table 3:** Sorafenib-related adverse events, treatment response and overall survival

Variable		Count	Percentage
Ascites		23	30.7%
Elevated liver enzymes		15	20%
Hematemesis		5	6.7%
Hand-foot skin reaction		5	6.7%
Hepatic encephalopathy		3	4%
Hypertension		2	2.7%
Arrhythmia		2	2.7%
Melena		1	1.3%
Jaundice		1	1.3%
Diarrhoea		1	1.3%
Anorexia		1	1.3%
Ischemic cardiomyopathy		1	1.3%
Acute kidney injury		1	1.3%
Severe hypoglycaemia		1	1.3%
Performance status deterioration		1	1.3%
Final modified RECIST	Not assessed	32	42.7%
	Partial response	1	1.3%
	Progressive disease	29	38.7%
	Stable disease	13	17.3%
Survival	Alive	9	12.3%
	Died	64	87.7%
		**Median**	**Range**
Survival time in Months Median (Min-Max)		4	1–20
Post sorafenib survival Median (Min-Max)		1.5	0–15

In the current study, 58.67% of the patients were sorafenib-naïve, while 41.33% were preceded by local/locoregional therapy. TACE was the most common modality, in 34.67%, followed by TARE in 2.67% of the patients (Table 4). Accordingly, we divided our patients into two groups: Group 1: Sorafenib-naïve patients (*n* = 44), and Group 2: patients with previous local/locoregional therapy (*n* = 31). There were no statistically significant differences between the two groups regarding demographic features. However, AST was significantly higher in sorafenib-naïve patients compared to patients with previous local/locoregional therapy (median = 51 versus 42, p-value = 0.03). Also, there was a significant difference between the two groups in terms of ALT, being slightly higher in sorafenib-naïve patients (median = 33 versus 28, p-value = 0.04). Other laboratory parameters showed no distinct difference between the two groups. Regarding HCC characteristics, sorafenib-naïve patients were more likely to have mild ascites (p-value = 0.04). Portal invasion was more prevalent in sorafenib-naïve patients (p-value = 0.008). In addition, sorafenib-naïve patients were more likely to have the more advanced BCLC-C stage (p-value < 0.001) (Table 5).

**Table 4:** Interventional therapy prior to sorafenib intake

Variable		Count	Percentage
Previous local/ locoregional therapy	TACE	26	34.67%
	TARE	2	2.67%
	Resection + MWA	1	1.33%
	TACE + MWA	1	1.33%
	MWA	1	1.33%
No		44	58.67%

**Table 5:** Comparison of demographic, laboratory, and radiologic features of sorafenib-naïve versus previously treated patients

Variable				Sorafenib naïve patients (*n* = 44)		Previously treated patients (*n* = 31)		*P* value
				Mean	SD	Mean	SD	
Age				60	± 10	63	± 7	0.18
				**Count**	**Percentage**	**Count**	**Percentage**	
Sex		Male		33	75%	23	74.2%	1
		female		11	25%	8	25.8%	
Smoker				13	29.5%	11	35.5%	0.62
Diabetes mellitus				6	13.6%	7	22.6%	0.36
HCC family history				2	4.5%	1	3.2%	1
				**Mean**	**SD**	**Mean**	**SD**	
Haemoglobin				12.45	± 2.00	12.65	± 2.06	0.67
Neutrophil%				65.23	± 9.83	64.11	± 7.47	0.6
Lymphocyte %				26.19	± 8.35	28.97	± 7.47	0.1
Total Bilirubin				0.94	± 0.43	1.02	± 0.48	0.8
Albumin				3.74	± 0.51	3.79	± 0.56	0.7
				**Median**	**Range**	**Median**	**Range**	
WBCs				5.95	2.9–13.20	6.7	2.7–11.7	0.97
Platelets				170	64–375	146	55–353	0.154
CRP				15.65	7.45–53.45	5.5	2.3–11.3	0.27
ALT				33	24.-60	28	24–42	**0.04**
AST				51	36.75	42	29–52	**0.03**
INR				1.14	1.03–1.26	1.19	1-1.13	0.6
AFP				802	102–3283	203	47-1210	0.2
				**Count**	**Percentage**	**Count**	**Percentage**	
Mild ascites				6	13.6%	0	0.0%	**0.04**
Portal vein	Patent			18	40.9%	23	74.2%	**0.008**
	Thrombosed			26	59.1%	8	25.8%	
Lymph nodes involvement				24	54.5%	12	38.7%	0.24
Distant Metastases				9	23.1%	4	13.3%	0.37
Number of hepatic focal lesions			**1**	20	45.5%	13	41.9%	0.32
			**2**	2	4.5%	5	16.1%	
			Multiple	21	47.7%	13	41.9%	
Site of lesion(s)			Right lobe	23	53.3%	17	54.8%	0.45
			Left lobe	7	16.3%	2	5.5%	
			Both lobes	13	30.2%	12	38.7	
BCLC stage			B	2	4.5%	14	45.2%	**< 0.001**
			C	42	95.5%	17	54.8%	

*WBCs* white blood cells, *ALT* alanine transaminase, *AST* aspartate transaminase, *INR* international normalized ratio, *AFP* alpha-feto protein, *MELD score* model for end stage liver disease, *BCLC* Barcelona Clinic Liver Cancer

As shown in Table 6, there were no statistically significant differences between the two groups in terms of neutrophil-lymphocyte ratio, albumin-to-bilirubin grade, Glasgow prognostic score, or CRAFITY score.

**Table 6:** Pretreatment predictive scores of sorafenib-naïve versus previously treated patients

Variable		Sorafenib naïve patients (*n* = 44)		Previously treated patients (*n* = 31)		P value
		Count	percentage	count	percentage	
Neutrophil to lymphocyte ratio	Low	32	76.2%	26	83.9%	0.56
	High	10	23.8%	5	16.1	
ALBI grade	1	12	27.3%	13	41.9%	0.32
	2	31	70.5%	18	58.1%	
	3	1	2.3%	0	0.0%	
CRAFITY score	low	5	11.4%	9	30%	0.14
	Intermediate	18	40.9%	11	36.7%	
	High	21	47.7%	10	33.3%	
Glasgow prognostic score	0	13	29.5%	12	40.0%	0.64
	1	23	52.3%	13	43.3%	
	2	8	18.2%	5	16.7%	

There were no statistically significant differences between the two groups in relation to various sorafenib adverse events, treatment response according to modified RECIST, or in terms of survival (Table 7).

**Table 7:** Sorafenib- related adverse events, treatment response and survival of sorafenib-naïve versus previously treated patients

Variable		Sorafenib naïve patients (*n* = 44)		Previously treated patients (*n* = 31)		*P* value
		Count	Percentage	Count	Percentage	
Ascites		13	29.5%	10	32%	1
Elevated liver enzymes		8	18.2%	7	22.6%	0.77
Hematemesis		3	6.8%	2	6.5%	1
Melena		0	0.0%	1	3.2%	0.41
Jaundice		0	0.0%	1	3.2%	0.41
Hepatic encephalopathy		3	6.8%	0	0.0%	0.26
Diarrhea		0	0.0%	1	3.2%	0.41
Anorexia		1	2.3%	0	0.0%	1
Hand-foot skin reaction		3	6.8%	2	6.5%	1
Hypertension		1	2.3%	1	3.2%	0.77
Ischemic cardiomyopathy		1	2.3%	0	0.0%	1
Arrhythmia		0	0.0%	2	6.5%	0.17
Acute kidney injury		1	2.3%	0	0.0%	1
Severe hypoglycaemia		1	2.3%	0	0.0%	1
Performance status deterioration		1	2.3%	0	0.0%	0.41
Survival	Alive	6	13.6%	3	10.3%	0.73
	Died	38	86.4%	26	89.7%	
The modified RECIST	Not assessed	22	50%	10	32.3%	
	Partial response	0	0.0%	1	3.2%	0.25
	Progressive disease	16	36.4%	13	41.9%	
	Stable disease	6	13.6%	7	22.6%	

As progressive and stable diseases were the most common treatment responses, we compared various parameters between the two response groups. No significant differences were observed between the two groups in terms of demographic characteristics, MELD score, BCLC stages, neutrophil-lymphocyte ratio, ALBI grade, or CRAFITY score (Table 8).

**Table 8:** Features of patients with stable and progressive diseases after Sorafenib treatment

Variable		Stable disease (*n* = 14)		Progressive disease (*n* = 29)		*P* value
		Median	Range	Median	Range	
Age		66	58–70	60	56–66	0.09
Neutrophil to lymphocyte ratio		2.06	1.58–3.13	2.52	1.91–2.96	0.4
ALBI grade		-2.42	-2.69- -2.02	-2.36	-2.86- -1.89	0.9
MELD score		8.5	6–11	9	8–11	0.7
		**Count**	**Percentage**	**Count**	**Percentage**	
Gender	Male	8	57.14%	24	82.76%	0.7
	Female	6	42.86%	5	17.24%	
Smoking history		3	21.42%	13	44.82%	0.1
Diabetes mellitus		4	28.5%	3	10.34%	0.1
Baseline PS	**0**	4	28.57%	12	41.37%	0.55
	**1**	9	64.28%	15	51.7%	
	**2**	1	7.14%	1	3.44%	
BCLC stage	B	7	50%	6	20.68%	0.05
	C	7	50%	23	79.32%	
CRAFITY score	Low	5	35.7%	7	24.13%	0.6
	Intermediate	5	35.71%	10	34.48%	
	High	4	28.57%	12	41.37%	

The overall survival analysis was conducted, and the Median overall survival was 4 Months (95% CI = 3.12–4.87). A time-to-progression analysis was conducted, and the median time to progression was 9 months.

The median overall survival was slightly higher in patients with grade 1 ALBI and among patients with a low CRAFITY score (7 months) when compared to intermediate and high scores (4 months), but without statistical significance. However, there were no statistically significant differences in terms of overall survival between patients with low versus high neutrophil-to-lymphocyte ratio or various Glasgow prognostic scores. However, (Fig. 1).

**Fig. 2:**
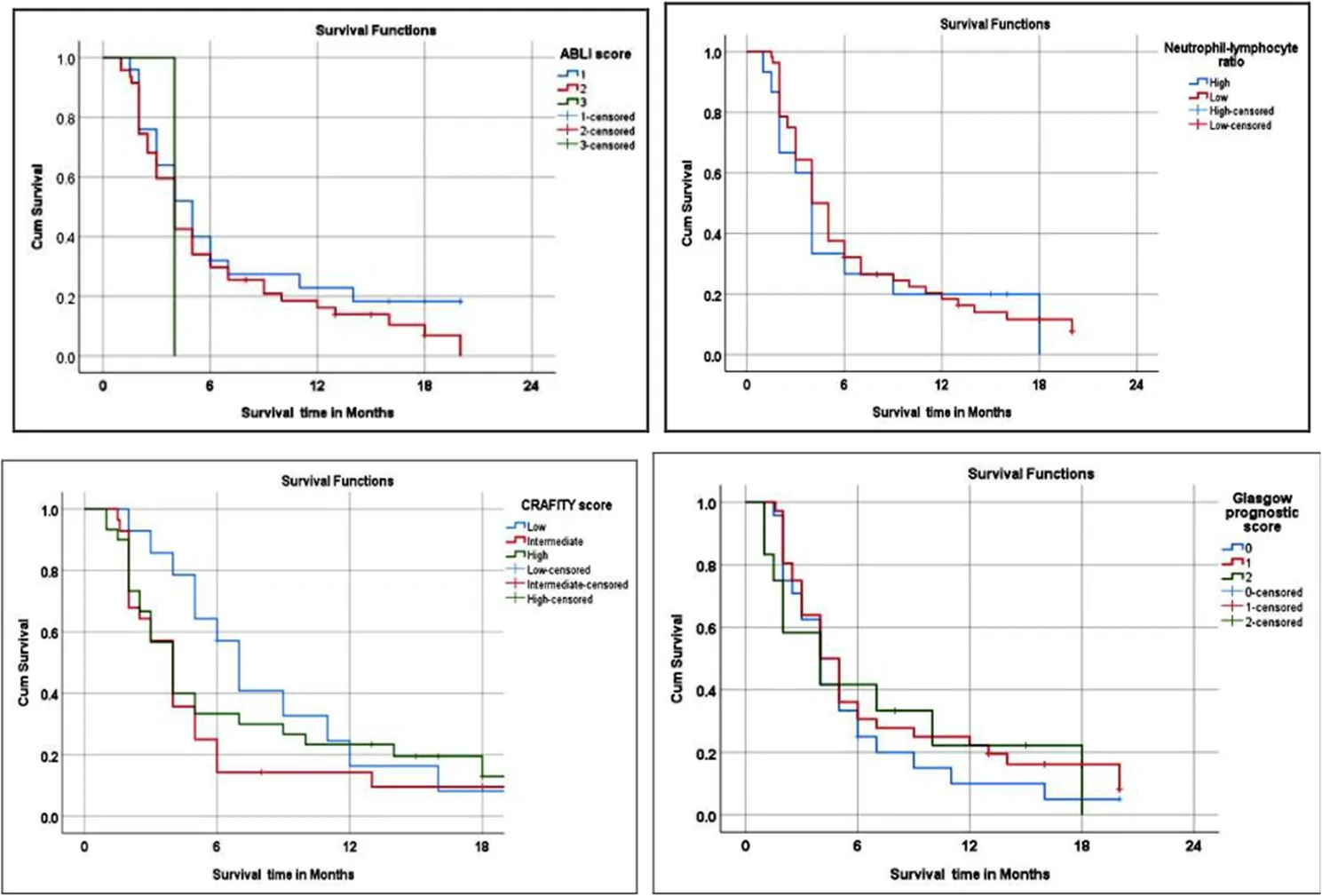
The relation between the overall survival and the studied scores

As shown in Fig. 2, the time to progression was slightly higher in patients with a high neutrophil-to-lymphocyte ratio when compared with a low ratio, but without significant differences. However, there were no significant differences in median time to progression in patients with various ALBI grades, various CRAFITY scores, or various Glasgow prognostic scores.

Univariate analysis showed that, relative to those who did not develop any side effects, those who developed side effects have an increased risk of death at all times of follow-up (HR: 2.33, 95% CI: 1.23–4.42). This was statistically significant, *p* = 0.01(Table 18) (Table 9). Multivariate analysis showed that advanced age was significantly correlated with less hepatocellular carcinoma progression, p-value = 0.04 (HR: 0.97, 95% CI: 0.93–0.99) (Table 10).

**Table 9:** Univariate COX regression for overall survival

Variable	Hazard ratio (95% CI)	*P*-value
Age	1.01 (0.99–1.04)	0.4
Gender Female	0.96 (0.55–1.68)	0.88
Smoking	0.66 (0.38–1.15)	0.1
DM	1.12 (0.58–2.16)	0.7
Baseline BCLC C vs. B	1.52 (0.81–2.86)	0.2
Size of HCC (largest diameter**)**	0.96 (0.89–1.04)	0.3
HFL number	0.89 (0.69–1.16)	0.4
PVT	1.56 (0.95–2.55)	0.08
ALBI score	1.29 (0.78–2.13)	0.3
Neutrophil to lymphocyte ratio	1.04 (0.86–1.26)	0.7
MELD	1.07 (0.97–1.19)	0.2
PS	0.99 (0.63–1.56)	0.9
Side effects		
Overall	2.33 (1.23–4.42)	**0.01**
HFS	1.03 (0.41–2.58)	0.9
Elevated liver enzymes	1.21 (0.66–2.21)	0.5
Ascites	0.99 (0.59–1.68)	0.99
Hepatic encephalopathy	1.93 (0.60–6.22)	0.3

**Table 10:** Multivariate Cox regression analysis for predictors of HCC progression

	Hazard ratio (95% CI)	*P*-value
Age	0.97 (0.93–0.99)	0.04
Gender Female	0.39 (0.14–1.14)	0.08
Smoking	1.63 (0.75–3.54)	0.2
DM	0.34 (0.08–1.44)	0.1
Baseline BCLC C vs. B	1.70 (0.67–4.31)	0.3
Size of HCC (largest diameter)	0.98 (0.87–1.11)	0.8
ALBI score	0.84 (0.39–1.78)	0.6
Neutrophil to lymphocyte ratio	0.95 (0.71–1.27)	0.7
MELD	1.07 (0.92–1.25)	0.4
PS	0.77 (0.37–1.61)	0.5
Duration of treatment		
Side effects		
Overall	0.65 (0.30–1.43)	0.3
HFS	1.06 (0.25–4.53)	0.9
Elevated liver enzymes	0.69 (0.24–2.02)	0.5
Ascites	0.99 (0.45–2.21)	0.99
Hepatic encephalopathy	1.85 (0.24–13.97)	0.55

## Discussion

Although diagnostic and screening methods have evolved, most HCC patients are found to have advanced disease (BCLC-C), making them ineligible for locoregional or surgical interventions [4]. Although sorafenib is currently among the recommended first-line medications for patients with BCLC stage C HCC, response to sorafenib treatment remains unpredictable despite efforts to define prognosis by clinical stages [10].

**Fig. 3:**
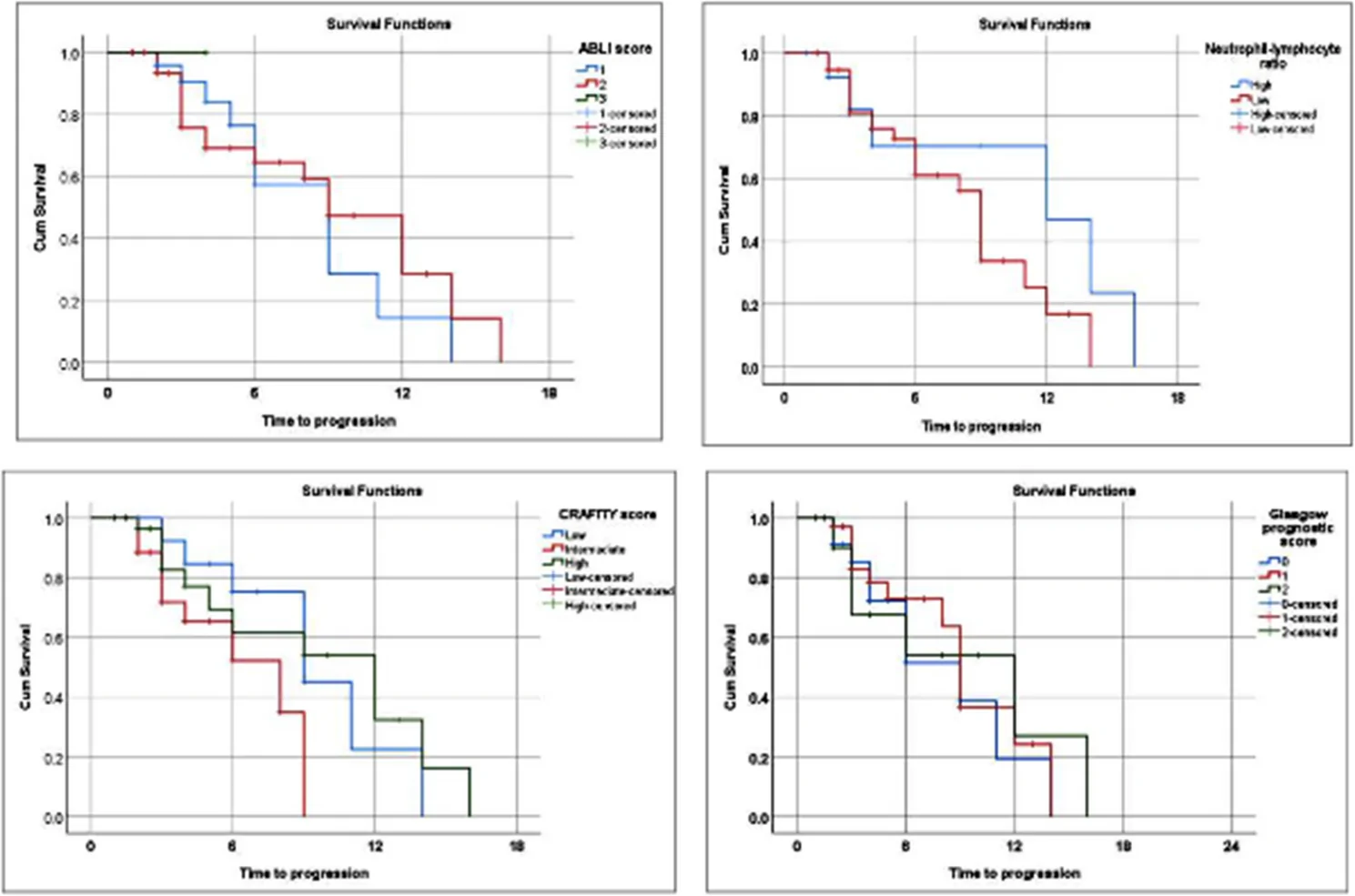
The relation between time to progression and the studied scores

Several studies showed that elevated baseline neutrophil-to-lymphocyte ratio, ALBI grade, CRAFITY score, and Glasgow prognostic score were significantly associated with worse overall survival in HCC patients treated with Sorafenib [13–16]. This study aims to predict treatment response and survival in patients with sorafenib-treated HCC using neutrophil-to-lymphocyte ratio, albumin-bilirubin grade, CRAFITY score, and Glasgow prognostic score (Fig. 3).

Seventy-five Egyptian patients with HCC, tumor progression after TACE, and advanced-stage HCC after treatment with sorafenib who attended the HCC multidisciplinary clinic at Kasr Al-Ainy Hospital, Cairo University, were enrolled into our study. Pretreatment laboratory parameters and imaging studies were obtained. Neutrophil to lymphocytes ratio, ALBI grade, CRAFITY score, and Glasgow prognostic score were calculated for each patient. All the patients were followed for treatment response, survival, and sorafenib intolerance.

In the current study, most of the participants were males, contributing around (74.67%). This result agreed with the study by Rashed et al., which showed that HCC is more common among Egyptians males [22]. This could be attributed to higher occupational exposure to viral hepatitis in men than women and, in addition, to the possible effect of androgens. The median age of the study patients was 62 years. Our result was consistent with Elkenawy et al., who showed that the incidence of HCC was higher in elderly patients, specifically more than 50 years [23]. This could be explained by the long duration of liver cirrhosis that predisposes to HCC development.

In our study, HCV was the most common underlying cause of liver cirrhosis and HCC. Our results was consistent with Omar et al., study, which demonstrated that HCV was the most common cause of HCC in Egypt [24]. Egypt had the highest burden of HCC worldwide. The median time to HCC development was 36 months; this result was comparable to Tani et al.., study, which showed that the incidence of HCC after DAA therapy was 1.88% after one year and 6.00% after 36 months [25]. In contrast, Conti et al., showed that HCC developed within the first 6 months post-DAAs treatment [26]. This could be explained by different follow-up intervals in different studies.

Our study demonstrated that Child-Pugh score A5 was the most common, 42 patients, followed by A6, 31 patients. This result was consistent with the GIDEON study, which showed that the overall survival was better in Child-Pugh score A than B [27]. Sorafenib therapy should be used in patients with preserved liver function to optimize the outcome and to decrease drug intolerance.

The current study showed that multiple hepatic focal lesions were predominant, and the right lobe was involved in most of our studied patients. This result was concordant with El Fayoumie et al.‘s study, which showed that HCC post-DAA therapy was multifocal and infiltrative [28]. Abdelaziz et al. showed that HCC behavior was more aggressive in DAA-treated patients regarding portal vein thrombosis, malignant lymphadenopathy, and HCC imaging characteristics [29]. In addition, Kumar et al. Showed that the right lobe was the most common site of HCC involvement [30]. Portal vein invasion was detected in 45.33%, lymph node involvement in 48% and tumor metastases in 18.84% of the studied population. This result was comparable to El Fayoumie et al. study, which demonstrated a higher incidence of portal invasion and nodal metastases in patients with advanced HCC after DAAs [27]. This could be explained by the aggressive behavior of HCC after DAA therapy and by the advanced stage of HCC.

Our study showed that most of the patients had a low pretreatment neutrophil-to-lymphocyte ratio (77.33%), grade 2 ALBI (65.33%), 41.89% had a high CRAFITY score, and 48.6% had Glasgow prognostic score 1. This result was comparable to the Fidan et al. study, which demonstrated that most of the treated patients had a low neutrophil-to-lymphocyte ratio and grade 2 ALBI [31]. However, our results were contradictory to Zhang et al., which showed that high CRAFITY scores were detected in 22.81% of the patients [32]. In the current study, 58.67% of the patients were sorafenib-naïve, while 41.33% were preceded by local/locoregional therapy; sorafenib was prescribed for HCC progression. Accordingly, we divided our patients into two groups: Group 1: sorafenib-naïve patients (*n* = 44), and Group 2: patients with previous local/locoregional therapy (*n* = 31).

Our study demonstrated that pretreatment AST and ALT levels were significantly higher in patients with in sorafenib naïve patients in compared to patients with previous local/locoregional therapy. This could be explained by the advanced stage of liver cirrhosis upon which HCC developed; in addition, more advanced-stage HCC in sorafenib naïve.

Our study showed no significant statistical differences between the two groups regarding patient characteristics, age, gender, underlying cause, or most of the laboratory parameters, except for AST and ALT. This result was consistent with Kimura et al. study, which demonstrated no significant statistical correlation with age, sex, underlying cause, Child score, or laboratory parameters [33].

The current study demonstrated that mild ascites, portal invasion, and BCLC stage C were significantly higher among sorafenib-naïve patients when compared to previously treated, local/loco-regional patients. In contrast, the Wu et al. study showed no significant difference between the two groups in terms of ascites, portal invasion, and BCLC stages [34]. Most of the patients in our study belong to BCLC stage C, so this advanced presentation could contribute to a higher risk of portal invasion and hepatic decompensation.

Most of the studied patients developed adverse events to sorafenib. Hepatic decompensation by ascites was the most common type of intolerance, followed by elevated liver enzymes, HFSR, and hematemesis. This result was comparable to the Raoul et al. study, which showed that sorafenib adverse events were seen in 73% of the patients. Unlike our study, dermatological side effects were the most common, followed by hepatic adverse events [35]. This could be related to higher tumor burden in our patients and to higher tumor aggressiveness, as most of our patients developed HCC after treatment with direct-acting antiviral agents (DAAs therapy.

Regarding the treatment response, progressive disease was the most common response, followed by stable disease. Similar results were obtained by Wu et al., who showed that progressive disease was the most common treatment response. There were no statistically significant differences between the two groups [34]. A contradictory result was obtained by Ramadan et al., who reported that a stationary course was higher in patients with advanced HCC after treatment with sorafenib [36]. Our study showed that 87.7% of the patients had died by the end of the study. The median overall survival was 4 months. The current result was comparable to the Nada et al. study, which demonstrated that the median overall survival of patients with hepatocellular carcinoma after sorafenib therapy was 5 months [37]. In contrast, a meta-analysis showed a higher survival rate than our study [38]. The time to radiological progression in our study was 9 months. El Baghdady et al. study showed that the TTP was 12 months. In contrast, Lopez-Lopez et al. study demonstrated that the time to progression was 4.5 months [39]. This conflict results could be explained by different sample sizes in various studies. There were no significant differences between the two groups in terms of time to progression. This result was consistent with Zhang et al. study, which showed no significant differences [40].

The current study showed insignificant differences in the overall survival and time to progression in patients with low versus high pretreatment neutrophil to lymphocyte ratio. Contradictory to our results, Casadei-Gardini et al. demonstrated that patients with low pretreatment neutrophil-to-lymphocyte ratio had significantly increased overall survival and TTT when compared to higher pretreatment NLR [41].

In our study, the overall survival and time to progression were not statistically insignificant among various ALBI grades. This result was contradictory to the Ogasawara et al. study, which showed significant differences between ALBI grade 1 and 2 in terms of overall survival [42]. In addition, Kuo et al., showed higher OS and TTP for ALBI grade 1 when compared to ALBI grades 2 and 3 [14].

The overall survival in our study was higher in patients with a low CRAFITY score but without statistical significance when compared to intermediate and high scores. The time to progression also was insignificant between various CRAFITY grades. Scheiner et al. study demonstrated significantly higher overall survival among patients with a low CRAFITY score when compared to higher ones [15]. The relatively small sample size in our study could be the cause of such conflicting results.

In our study, the overall survival and time to progression were not statistically significant among various Glasgow prognostic scores. Contradictory to our result, Morimoto et al., showed higher OS and TTT for GPS 0 when compared to scores 1 and 2 [16].

Sorafenib adverse events, hand-foot skin reaction and diarrhea, were significantly correlated with increased overall survival [43–44]. In contrast, our study showed a significantly higher mortality rate among patients who developed adverse events to sorafenib. This could be attributed to different types of sorafenib intolerance in our cohort; ascites and hepatotoxicity were the commonest events, in contrast to HFSR and diarrhea in other studies. Unexplainedly, advanced age was significantly correlated with less hepatocellular carcinoma progression in our study. The advent and availability of effective and well-tolerated combined immunotherapy for patients with advanced hepatocellular carcinoma has resulted in limitation of sorafenib provision for such population.

In conclusion, Overall survival and time to progression are not affected by different pretreatment values of NLR, ALBI grade, CRAFITY score, and Glasgow prognostic score in sorafenib-treated HCC. This contradiction could be explained by a relatively small sample size, tumor characteristics such as highly aggressive post-DAAs HCC, more advanced stage with portal invasion, and could be due to drug intolerance.

## Data Availability

No datasets were generated or analysed during the current study.
